# Molecular breeding of a novel orange-brown tomato fruit with enhanced beta-carotene and chlorophyll accumulation

**DOI:** 10.1186/s41065-016-0023-z

**Published:** 2017-01-11

**Authors:** Ranjith Kumar Manoharan, Hee-Jeong Jung, Indeok Hwang, Namhee Jeong, Kang Hee Kho, Mi-Young Chung, Ill-Sup Nou

**Affiliations:** 1 Department of Horticulture, Sunchon National University, 255, Jungang-ro, Suncheon, Jeonnam 57922 Republic of Korea; 2 Department of Fisheries Science, Chonnam National University, 50, Daehak-ro, Yeosu, Jeonnam 59626 Republic of Korea; 3 Department of Agricultural Education, Sunchon National University, 255, Jungang-ro, Suncheon, Jeonnam 57922 Republic of Korea; 4 Present address: Department of Horticulture, Sunchon National University, 255 Jungang-ro, Suncheon, Jeonnam 57922 Republic of Korea

**Keywords:** Orange-brown tomato, *CYC-B*, β-carotene, *SGR*, Chlorophyll

## Abstract

**Background:**

Tomatoes provide a significant dietary source of the carotenoids, lycopene and β-carotene. During ripening, carotenoid accumulation determines the fruit colors while chlorophyll degradation. These traits have been, and continue to be, a significant focus for plant breeding efforts. Previous work has found strong evidence for a relationship between *CYC-B* gene expression and the orange color of fleshy fruit. Other work has identified a point mutation in *SGR* that impedes chlorophyll degradation and causes brown flesh color to be retained in some tomato varieties.

**Methods:**

We crossed two inbred lines, KNY2 (orange) and KNB1 (brown) and evaluated the relationship between these genes for their effect on fruit color. Phenotypes of F2 generation plants were analyzed and a novel ‘orange-brown’ fruit color was identified.

**Results:**

We confirm two SNPs, one in *CYC-B* and another in *SGR* gene sequence, associated with segregation of ‘orange-brown’ fruit color in F2 generation. The carotenoid and chlorophyll content of a fleshy fruit was assessed across the different phenotypes and showed a strong correlation with expression pattern of carotenoid biosynthesis genes and SGR function. The orange-brown fruit has high β-carotene and chlorophyll. Our results provide valuable information for breeders to develop tomato fruit of a novel color using molecular markers.

**Electronic supplementary material:**

The online version of this article (doi:10.1186/s41065-016-0023-z) contains supplementary material, which is available to authorized users.

## Background

Tomatoes are predominantly grown as an agricultural crop and are considered a healthy food due to their high nutritional value [1]. Tomatoes are cholesterol free, rich in fiber and protein, and low in fat and calories. Approximately 80% of the tomatoes produced are used in tomato-based foods that include tomato juice, puree, paste, sauce and salsa [2]. Daily consumption of tomato sauce has been shown to reduce DNA damage in white blood cells and cancerous prostate tissues [3]. In addition, consumption of lycopene-containing foods can reduce the risk of cardiovascular disease and breast cancer [4]. Therefore, many attempts have been made to develop high-lycopene tomatoes using conventional breeding techniques and genetic manipulation. In addition to lycopene content, new color developments also attract consumers in the fresh market. Tomato fruit color is an important indicator of eating quality for consumers and thus considerable research has been directed towards its characterization and measurement [5]. During the ripening stage, tomato color brightens due to carotenoid, lycopene accumulation, independently or in concert with chlorophyll degradation [6]. Subsequent lycopene accumulation during the final stages of fruit ripening affects color development and the health benefits, both important traits for consumers. In green vegetables and leaves, lycopene is concealed by green chlorophyllic pigments. However, in most fruits, lycopene and other carotenoids are responsible for the bright color development during the ripening stage [7]. Lycopene accounts for more than 80% of the accumulated carotenes in ripe tomato fruits. While β-carotene accumulates to a lesser degree, it also constitutes a sizable portion of total carotene accumulation. Both lycopene and β-carotene are essential to fulfill the nutritional requirements of a healthy animal and human diet [8].

**Fig. 1:**
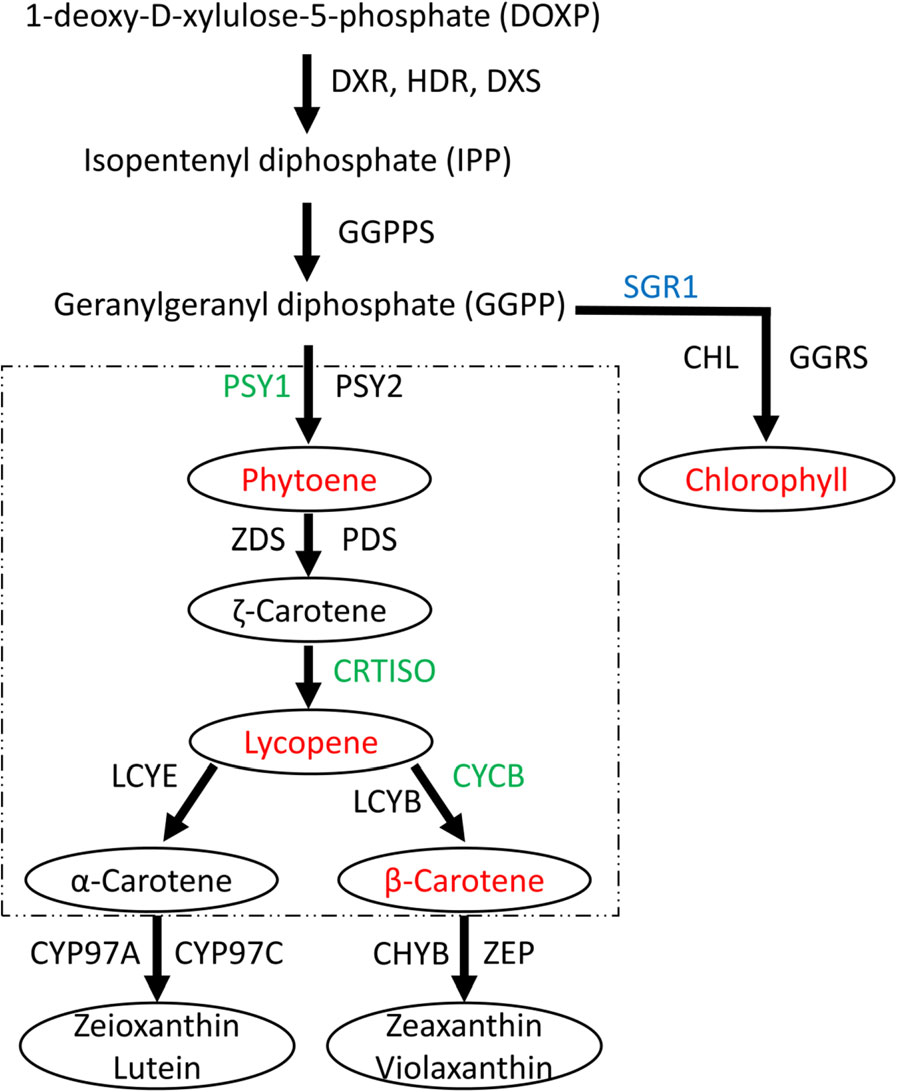
Metabolic pathways involved in biosynthesis of carotenoids via DOXP route with related genes of tomato. The enzymes involved in the carotenoid synthesis are: DOXP reductoisomerase (DXR), hydroxymethylbutenyl diphosphate synthase (HDS), hydroxymethylbutenyl diphosphate reductase (HDR), geranylgeranyl pyrophosphate synthase (GGPPS), phytoene synthase (PSY), phytoene desaturase (PDS), z-carotene desaturase (ZDS), carotene isomerase (CRTISO), lycopene ε cyclase (LCYE), lycopene β cyclase (LCYB), β-carotene hydroxylase (CHYB), cytochrome P450-type monooxygenase 97C (CYP97C), zeaxanthin epoxidase (ZEP), geranylgeranyl oxidase (GGRS), chlorophyll synthase (CHL). The carotenoids measured by HPLC are denoted in *red* color. Dotted rectangle denote carotenoid pathway, genes analyzed in this work are colored in *green* and regulatory gene SGR is colored in *blue*

Carotenoid biosynthesis in plants occurs preferentially via the 1-deoxy-D-xylulose-5-phosphate (DOXP) pathway rather than the previously assumed mevalonic acid pathway [9, 10]. Although both pathways produce isopentenyl pyrophosphate (IPP), the DOXP pathway [11] also leads to the formation of the plastidic carotenoids phytol, plastoquinone-9, and diterpenes (Fig. 1). IPP is a precursor of geranylgeranyl pyrophosphate (GGPP), which is a carotenoid precursor. Using phytoene synthase (*PSY*) as a catalyst with GGPP leads to production of phytoene [12, 13]. The phytoene desaturase gene (*PDS*) converts phytoene to ζ-carotene. Three isoforms of the *PSY* gene have been identified in tomato. The *PSY1* isoform is responsible for fruit ripening, whereas *PSY2* and *PSY3* are likely predominant in green tissues and roots, respectively [14–16]. *PSY1* and *PDS* genes are upregulated during fruit ripening, while genes for enzymes carrying out cyclisation of lycopene such as β-Lcy and ε-Lcy are downregulated, resulting in low levels of β-carotene during tomato ripening [17]. Our previous report had showed the evidence of *CYC-B* (*β-lycopene cyclase*) gene expression associated with orange color of fleshy fruit in tomato [16].

Another important gene, *SGR* (*STAY-GREEN*) associated with color has been identified previously in some plant species [18–20]. *SGR* mutants showed brown color due to carotenoid accumulation and fail to degrade chlorophyll completely at ripening stage [21, 22]. For instance, *SGR* mutants that have been identified in other plant species (Arabidopsis, pepper, pea, and meadow fescue) displayed green phenotypes due to inhibition of chlorophyll degradation [19, 23–27]. Barry and Pandey [30] had reported that point mutation in *SGR* gene causes loss of protein function and leads to inhibit chlorophyll degradation which exhibited green fleshy fruit color in ‘Black cherry’ variety. Flavonoids also play an important role in determining tomato fruit color. Flavonoids primarily accumulate in the tomato fruit peel, and are absent in the flesh, due to a lack of expression of flavonoid biosynthesis genes in flesh tissues [28, 29].

Because of their importance, our study focused on developing a new tomato fruit color that is enriched for β-carotene and chlorophyll content. Thus, our work evaluated the segregation of fruit color and, β-carotene and chlorophyll level, in the F1 and F2 populations, developed by crossing orange and brown fruit.

## Methods

### Plant material

Tomato seeds of inbred lines KNY2 (orange) and KNB1 (brown) were obtained from Kana Seed Co. Ltd (Korea). Seeds were sown on moist filter paper in petri dishes and germinated at 30 °C in the dark. The germinated seeds were transferred to plastic trays containing soil mix and maintained at growth room conditions. When plants (F1 and F2) had four true leaves, they were transplanted into plastic pots and grown in greenhouse (25 °C day/18 °C night, 70% air humidity and natural light) at Sunchon National University, Korea. Furthermore, three fruits were harvested from individual plant at ripe (57 days after pollination (DAP)) stage for HPLC analysis. Fruit color was confirmed from 6 F1 plants, 192 F2 plants (obtained from 6 F1 plants) and described in results section.

### RNA extraction and PCR amplification

Total RNA was extracted from fleshy fruit tissue at early (E, 17 DAP), mature (M, 39 DAP), turning (T, 45 DAP), and ripe (R, 57 DAP) stages using an RNA extraction kit (Qiagen, USA) according to manufacturer’s instructions. High-quality RNA was eluted in RNase-free water and treated with RNase-free DNase I (Qiagen) before cDNA synthesis. Quantitative RT-PCR (RT-qPCR) was conducted using cDNA synthesized from the RNA of each stage. PCR was performed with the following conditions: initial denaturation at 95 °C for 10 min, followed by 40 cycles of 95 °C for 20 s, 60 °C for 20 s, 72 °C for 40 s and final extension at 72 °C for 2 min.

### SNP detection

Single nucleotide polymorphisms (SNPs) were detected using 3’-blocked and unlabeled oligonucleotide probes (HybProbe). PCR was performed using LightCycler® 480 Resolight saturating dye (Roche, Germany) to generate melting curves characteristic of the genotype under the probe. Melting curves were generated and analyzed using the LightCycler®96 Instrument System (Roche, Germany). PCR reactions were performed with a 95 °C pre-denaturation for 5 min, followed by 45 cycles of denaturation at 95 °C for 20 s, annealing at 60 °C for 20 s, and extension at 72 °C for 30 s, with a final extension s at 72 °C for 40 s. Primer and probe sets used for SNP detection are described in Additional file 2 Table S1.

### HPLC analysis

Standards for β-carotene, lycopene, phytoene, and chlorophyll were purchased from Sigma- Aldrich (Sigma Co., USA). The carotenoids and chlorophyll were separated using reverse phase columns (Kinetex 206 μm, C18 100A, 100 × 4.60 mm, Phenomenex, USA). The whole fruit extracts were filtered with a 0.2 μm PTFE filter prior to injection. Mobile phase A was 78% methanol and B was 100% ethyl acetate. Release conditions were 0–5 min, 0% B; 5–15 min, 10% B; 15–20 min, 100% B; 20–30 min, 0% B at a flow rate of 1 mL/min. The phytoene, carotenoids and chlorophyll were identified and quantified based on the retention time and the absorbance between 280 nm, 450 nm and 660 nm of standards. The values represent the mean of three biological replicates.

### Statistical analysis

The *EF1α* gene was used as reference for normalization. The relative gene expression was calculated based on ΔΔCt method (LightCycler®96, Roche Diagnostics, Mannheim, Germany). Data are presented as the mean of three biological replicates. The data were analyzed using a Tukey Pairwise Comparisons test (*P* < 0.05) in the Minitab 17 Statistical Software (State College, Pennsylvania, USA). Chi-square analysis to test goodness-of-fit was performed using Graphpad Prism 7.02 (Graphpad software, California, USA).

## Results and discussion

### Segregation analysis

**Fig. 2:**
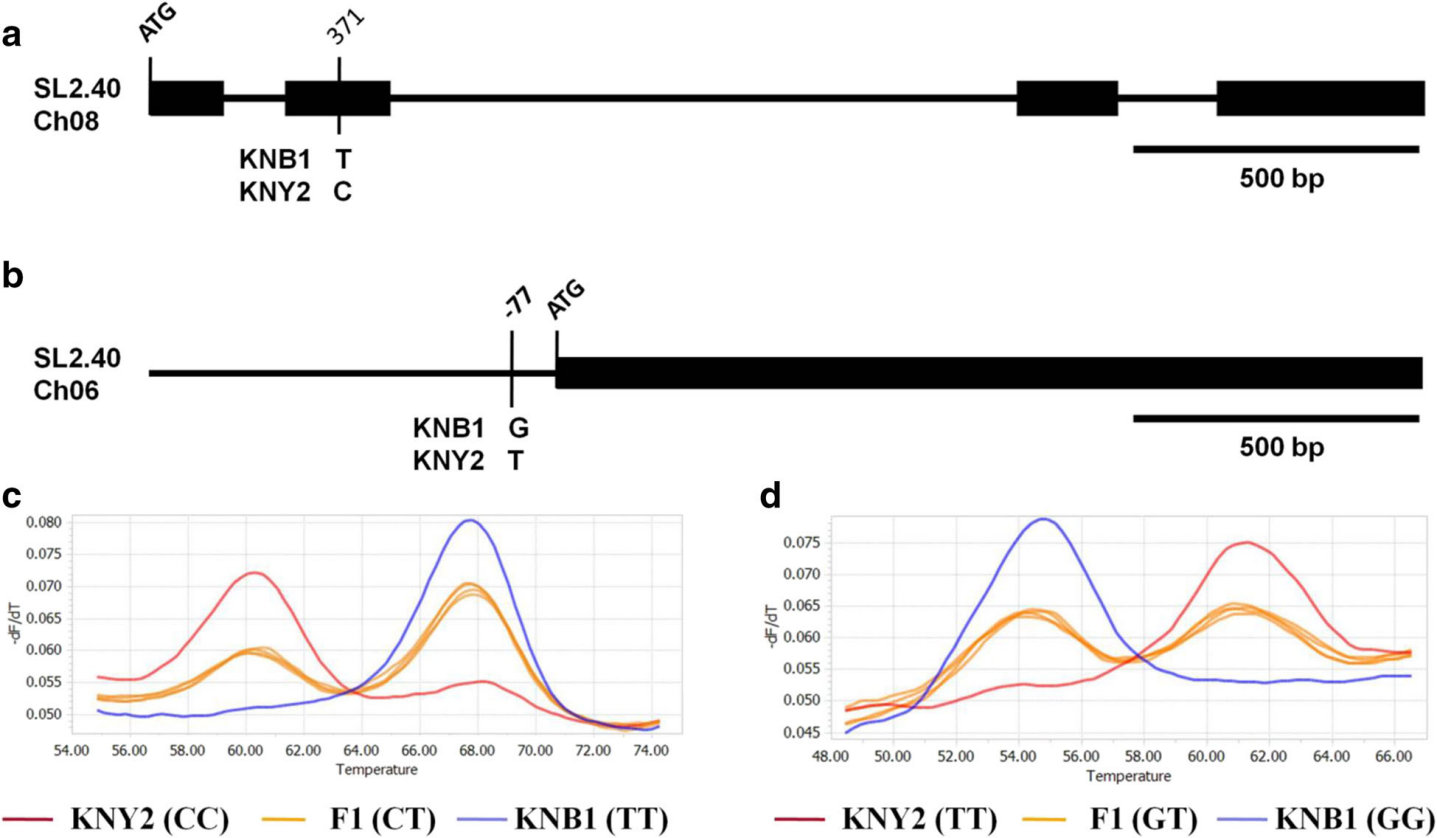
**a.** Structure of the *SGR* gene showing the SNP location 371 bp from the start codon as described by Barry and Pandey [30]. **b.** Promoter region of *CYC-B* gene identifying the SNP position at −77 as described by Hwang et al. [16]. **c.** SNP allele detection at *SGR* locus using Hybprobe with different melting curves. The genotypes include homozygous inbred parents KNY2 (CC, *red* melting curve) and KNB1 (TT, *blue* curve), and the heterozygous F1 (CT, *orange* curve). **d.** SNP detection in *CYC-B.* The genotype includes homozygous inbred parents KNY2 (TT, *red* melting curve) and KNB1 (GG, *blue* curve). The heterozygous F1 (GT) is represented with an *orange* curve

Inbred lines KNY2 and KNB1 were characterized for their fruit phenotype during ripening (Additional file 1: Fig. S1) and crossed. Fruit color phenotypes of F1 and F2 progeny were analyzed. All F1 progeny produced orange-colored fruit suggesting that orange color is dominantly inherited over other fruit colors in this experiment [16] (Additional file 1: Fig. S2). To examine the genotypic variation in the F2 generations, SNP sites of *SGR* and *CYC-B* genes were identified [16, 30]. The *SGR* gene plays an important role in the regulation of chlorophyll degradation in tomato fruits and leaves [31, 32]. The lack of SGR protein function in KNB1 is likely due to a C → T point mutation at position 371 bp from the start codon (ATG) in the *SGR* genomic sequence [30]. This modification results in brown-colored fruit, suggesting that chlorophyll is retained and conceals the pigmentation in the fruit (Fig. 2a, c). This observation confirms previous work by Barry and Pandey [30] identified this allele as responsible for the brown fruit color phenotype. Another gene that plays an important role in orange color formation and involved in improving carotenoid content in tomato fruit is *CYC-B* [33, 34]. This gene is suggested to play a major role in fruit pigmentation of the KNY2 inbred during the ripening stages. In a previous study, we detected and confirmed a SNP at the -77 position of the *CYC -B* gene that showed perfect association and a dominant phenotype in KNY2 for orange fruit color [16] (Fig. 2b, d). In the F1 generation, the 371 SNP site of *SGR* was detected as CT heterozygous which ‘T’ is a recessive allele (Fig. 2c). Similarly, the -77 SNP position of *CYC-B* was detected as GT heterozygous (Fig. 2d). These results suggest that a ‘T’ represents a dominant allele in *CYC-B* genes and leads to orange color formation in the F1 generation.

**Fig. 3:**
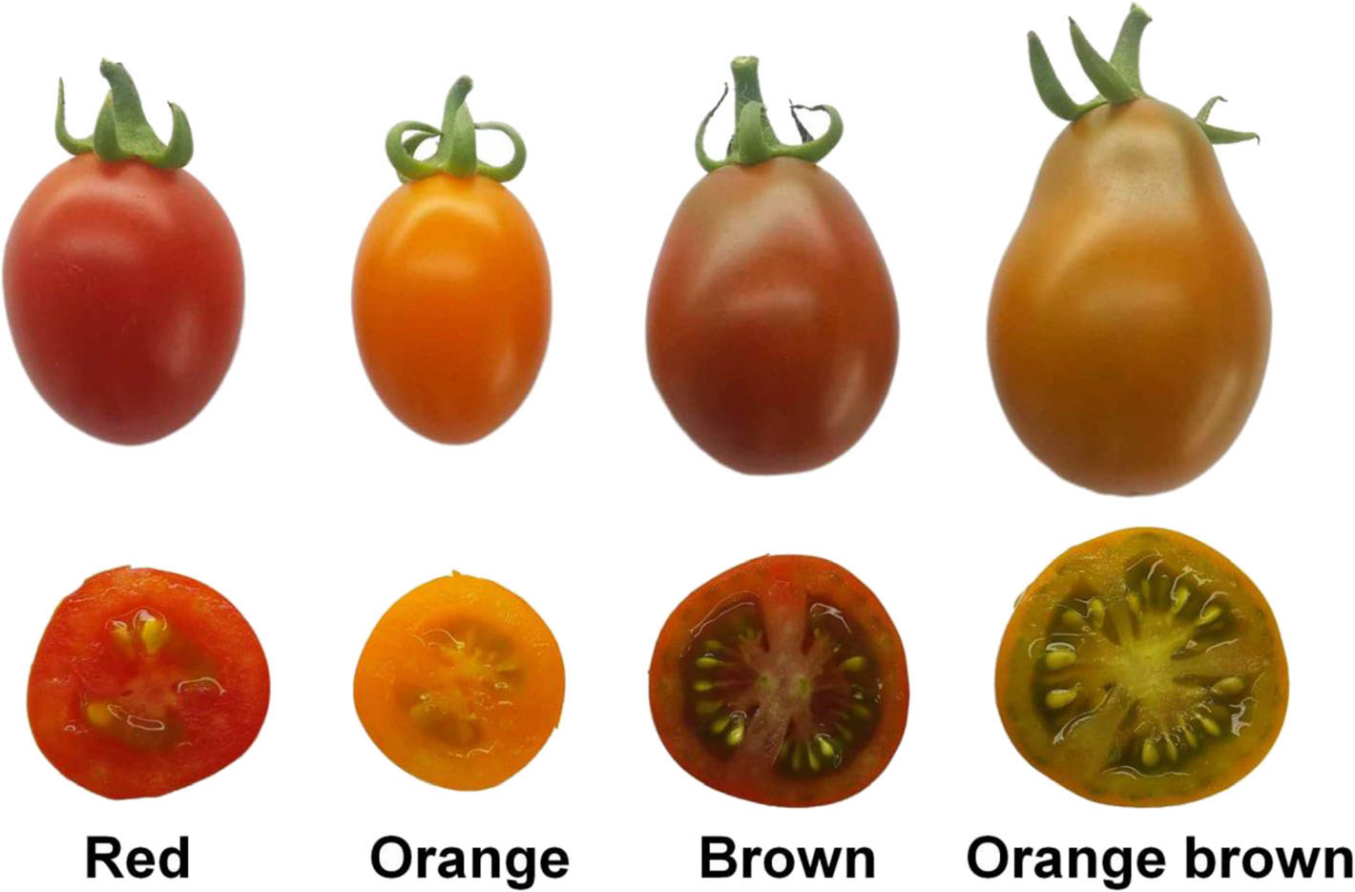
The fruit colors observed in the F2 generation at ripe stage (R, 57 DAP)

Fruit color phenotypes were scored in the F2 generation and the segregation ratios were analyzed with the corresponding genotypes. Four color phenotypes were observed in the F2 generation, namely red, orange, brown and orange-brown in the ripe stage (R, 57 DAP) (Fig. 3). Genotypes TT, CT and CC were identified at the 371 (*SGR*) SNP position whereas TT, GT and GG genotypes were identified at the -77 (*CYC-B*) SNP position (Table 1). In total, 192 F2 plants were evaluated (Table 1). Interestingly, a new ‘orange-brown’ color was identified in this F2 population and was found to segregate approximating a 9:3:3:1 (Orange: Red: Orange-brown: Brown) ratio (Table 2). The chi-square for F2 progeny segregation ratio showed an acceptable fit to 9:3:3:1 (Chi-square (*χ* ^2^) = 0.370, *P* >0.90). The genotype at the *SGR* 371 SNP site was confirmed to show approximating a 1:2:1 ratio of TT: CT: CC in the F2 plants. The F2 plants with a TT genotype were either orange-brown or brown in color, whereas the F2 plants with a genotype of CT and CC were either red or orange (Table 1). The *CYC-B -*77 SNP site with a TT or GT genotype corresponded with an orange or orange-brown phenotype while GG genotypes were either red or brown (Table 1). Fruit color was dependent on the genotypes at both the *SGR* and *CYC-B*.

**Table 1:** Phenotypic and genotypic segregation of F2 generation plants from KNB1 x KNY2

	No. Plants	Genotype		Phenotype	No. Plants
		*SGR* 371 position	*CYC-B* -77 position		
F1 generation	6	C-T	G-T	Orange	6
F2 generation	192	C-C	T-T	Orange	11
			G-T	Orange	22
			G-G	Red	18
		C-T	T-T	Orange	18
			G-T	Orange	56
			G-G	Red	21
		T-T	T-T	Orange-brown	12
			G-T	Orange-brown	22
			G-G	Brown	12

**Table 2:** Segregation ratio of fruit color in F2 generation plants from KNB1 x KNY2

Cross		F_2_ Segregation					*χ*2 (9:3:3:1)	*P*-value
		Orange	Red	Orange-brown	Brown	Total		
No. Plants	Observed	107	39	34	12	192	0.370	0.95-0.90
	Expected	108	36	36	12	192		

### Expression of *CYC-B* and *SGR*

**Fig. 4:**
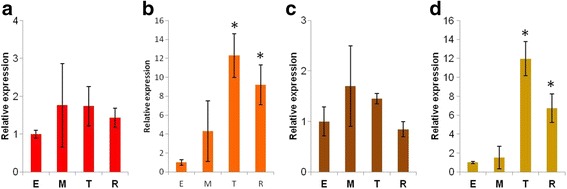
Expression of the *CYC-B* gene during fruit development in F2 generation plants. RNA was extracted from fruit samples at early (E), mature (M), turning (T), and ripe (R) developmental stages. Relative amounts of mRNA were determined by RT-qPCR after normalizing with *EF1α* transcript. The relative expression levels of *CYC-B* were compared to early stage (E) level (set to 1) in each F2 plant. Data represent an average ± s.e.m of three biological replicates and asterisk indicates values significantly different (*p* <0.05). **a**: *Red*, **b**: *Orange*, **c**: *Brown*, **d**: *Orange-brown*

The aim of this work was to identify the relationship between fruit color phenotype and variant genotypes in a segregating F2 population. Our previous work reported that the transcript level of *CYC-B* regulates orange color in tomato fruit [16]. Researchers focused on carotenoid content [17, 35] were also reported that regulation of *CYC-B* increases the accumulation of β-carotene in the tomato fruit during ripening. Similar results were observed during fruit development in bell pepper, a related species [36–39]. Based on the previous findings, we compared transcript levels of *CYC-B* among the different phenotypes observed in this experiment. The mRNA levels of *CYC-B* were analyzed during ripening at early, mature, turning and ripe stages as described by [40]. Interestingly, the orange-brown phenotype showed increased expression of *CYC-B* during fruit development, similar to that observed in the orange phenotype (Fig. 4). The level of *CYC-B* expression was significantly (Tukey Pairwise Comparisons test, p < 0.05) higher at the turning stage of ripening than in earlier stages in both phenotypes (Fig. 4). This corroborates our hypothesis that expression of *CYC-B* contributes to orange coloration as well as to the orange-brown color. As expected, the transcript level of *CYC-B* was not increased in red or brown phenotypes during ripening. The dramatic increase in expression of *CYC-B* during T and R stages of orange and orange-brown phenotypes suggests high β-carotene levels in both phenotypes. In addition to *CYC-B*, the expression of biosynthetic genes such as *PSY1,* carotenoid isomerase (*CRTISO*) and *SGR* was examined (Additional file 1: Fig. S3). The mRNA levels of these three genes showed a pattern of increased expression during ripening across all four phenotypes. No significant difference (Tukey Pairwise Comparisons test, *p* < 0.05) in gene expression was observed among the phenotypes during ripening (Additional file 1: Fig. S3).

Barry and Pandey [30] reported that a SNP (C → T) at nucleotide 371 in the tomato *SGR* gene results in a mutation that truncates the protein at glutamine 91. Characterized in the variety ‘Black Cherry,’ the truncation introduces a premature stop codon and is predicated to carry null alleles that cause complete loss of protein function. Varieties with this SNP could thus inhibit chlorophyll degradation and retain green flesh. Consistent with these results, the new orange-brown phenotype reported herein retains brown coloration may be due to loss of SGR protein function while concurrent high expression levels of *CYC-B* result in orange fruit color. This combination could explain the orange-brown phenotype we observed.

### HPLC analysis of carotenoid and chlorophyll content

We investigated the carotenoid and chlorophyll content of tomato fruit during various developmental stages of ripening. All phenotypes identified in the F2 population, red, orange, brown and orange-brown, were analyzed in M, T and R stages of ripening. Lycopene, β-carotene and phytoene levels were shown to increase as the fruit progressed through ripening stages for all four phenotypes (Table 3). The orange-brown phenotype was observed to have low levels of lycopene and high levels of β-carotene, similar to the orange phenotype in R stage (Table 3). This result was consistent with our transcript analysis showing that higher expression of *CYC-B* correlates with accumulation of β-carotene and causes orange color pigmentation [17] (Fig. 4). The high chlorophyll content after ripening of the orange-brown phenotype was similar in level to the brown phenotype (Table 3). By contrast, chlorophyll content was decreased in red and orange phenotypes at ripening, while there was no significant difference between brown and orange-brown phenotypes (*p* <0.05). Carotenoid and chlorophyll of brown tomato phenotypes (*SGR*) have positive effect on human health [41, 42]. Chlorophyll in tomato fruits combined with lycopene increased antioxidant activity [43] and protect cells against oxidants and electrophiles [44]. Chlorophyll in *SGR* mutant tomato fruits could reduce risk of colon cancer in humans [45] and their derivatives can be used as agents of photodynamic therapy in cancer [46]. *SGR* mutant varieties also have agronomical advantages, positive yield is recorded in maize *SGR* variety FS854 and *SGR* rice variety SNU-SG1 [47]. In addition, increased water, carbohydrates, and protein content in husks, cobs, and seeds are reported in *SGR* maize variety L087602. *SGR* mutant lines of wheat showed heat tolerance at terminal growth stage [48]. Therefore, it assumes, *SGR* mutant cultivars could be good source of better yield, fruit quality, and heat stress tolerance in tomato breeding. We found that retention of chlorophyll together with higher β-carotene produced ‘orange-brown’ phenotype of tomato.

**Table 3:** Phytoene, lycopene, β-carotene, and chlorophyll content in F2 generation plants. The carotenoid pigments were quantified using HPLC (μg/g fresh weight) (*n* = 3, ± s.e.m) during mature (M), turning (T), and ripe (R) stages of ripening

Selected plant in F2 generation	Stage	Phytoene	Lycopene	β-carotene	Chlorophyll (a+b)
Red	M	0.01±0.14	0.04±0.12	0.91±0.61	2.84±0.17
	T	0.46±0.42	14.03±0.02	2.97±0.35	0.14±0.38
	R	1.68±0.21	32.12±0.64	4.03±0.64	0.08±0.12
Orange	M	0.01±0.34	0.02±0.07	1.16±0.28	2.54±0.16
	T	0.18±0.13	0.16±0.27	7.94±2.32*	0.22±0.03
	R	0.21±0.15	0.58±0.45	11.07±1.61*	0.07±0.07
Brown	M	0.01±0.03	0.01±0.03	1.12±0.26	5.84±0.12
	T	0.24±0.02	8.27±0.28	1.84±0.17	2.97±1.06*
	R	0.56±0.14	27.64±0.17	2.62±0.13	2.05±0.57*
Orange-brown	M	0.01±0.07	0.13±0.16	1.03±0.57	3.27±0.16
	T	0.12±0.17	0.34±0.28	10.97±1.38*	2.68±0.26*
	R	0.18±0.03	0.46±0.31	11.74±2.28*	2.03±0.68*

Asterisk indicates phenotypes of which levels at same stages were significantly different than in the other phenotypes using a Tukey Pairwise Comparisons test (*p*<0.05)

## Conclusion

The present study developed a new tomato fruit color with an orange-brown phenotype. This fruit has high β-carotene content and retains chlorophyll through ripening. The expression of *CYC-B* mRNA coincided with the accumulation of β-carotene. The point mutation in *SGR* gene causes loss of protein function and leads to inhibit chlorophyll degradation. Present work provides insight into development of genotypes with enhanced β-carotene accumulation and chlorophyll retention in tomato fruits. Combination of these two SNPs would be suitable for breeding ‘orange-brown’ color tomato cultivars.

## Additional files

Additional file 1: Fig. S1. Phenotype of KNB1 and KNY2 tomato fruits at various fruit ripening stages. E, early stage; M, mature stage; T, turning stage; R, ripe stage. **Fig. S2.** Fruit color observed in the F1 generation of a cross between KNB1 and KNY2. **Fig. S3.** Relative expression of *PSY1* (A, D, G, J), *CRTISO* (B, E, H, K) and *SGR* (C, F, I, L) during fruit development of various inbred lines. Relative amounts of mRNA were determined by RT-qPCR after normalizing with *EF1α* transcript. Relative expression levels were compared to early stage (E) level (set to 1) in each F2 plant. Data represent an average ± s.e.m of three biological replicates and asterisk indicates values significantly different (*p* <0.05). A-C: red fruit, D-F: orange fruit, G-I: brown fruit, J-L: orange-brown fruit. E, early stage; M, mature stage; T, turning stage; R, ripe stage. (PPTX 413 kb)
Additional file 2: Table S1 Primers and probes used in this study. (DOCX 14 kb)
