# Supplementary figures and images for: Molecular breeding of a novel orange-brown tomato fruit with enhanced beta-carotene and chlorophyll accumulation
Source: Hereditas. 2017 Jan 11;154:1. doi: 10.1186/s41065-016-0023-z (PMC5226094; doi:10.1186/s41065-016-0023-z)

## Slide 1
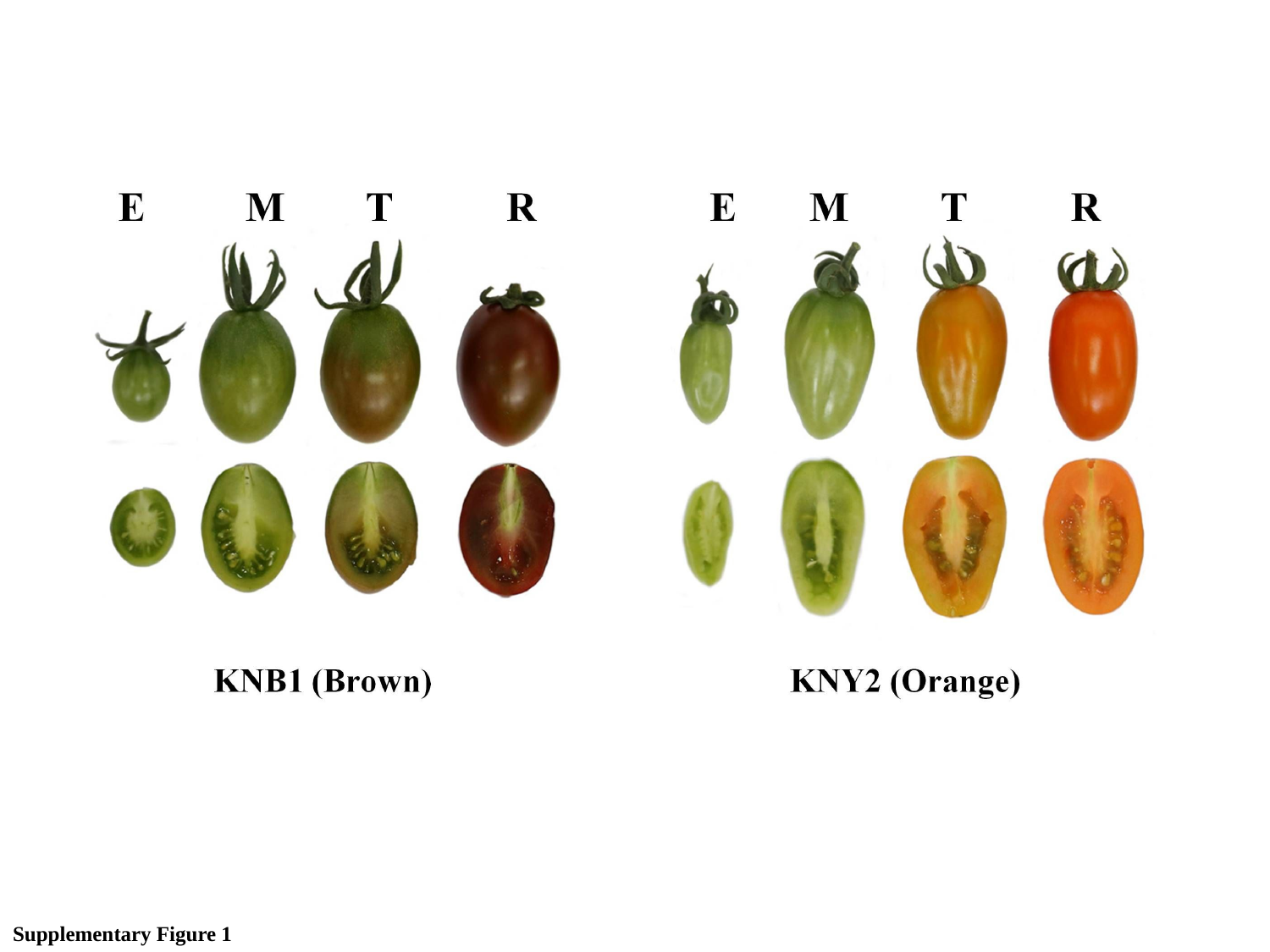

Supplementary Figure 1

## Slide 2
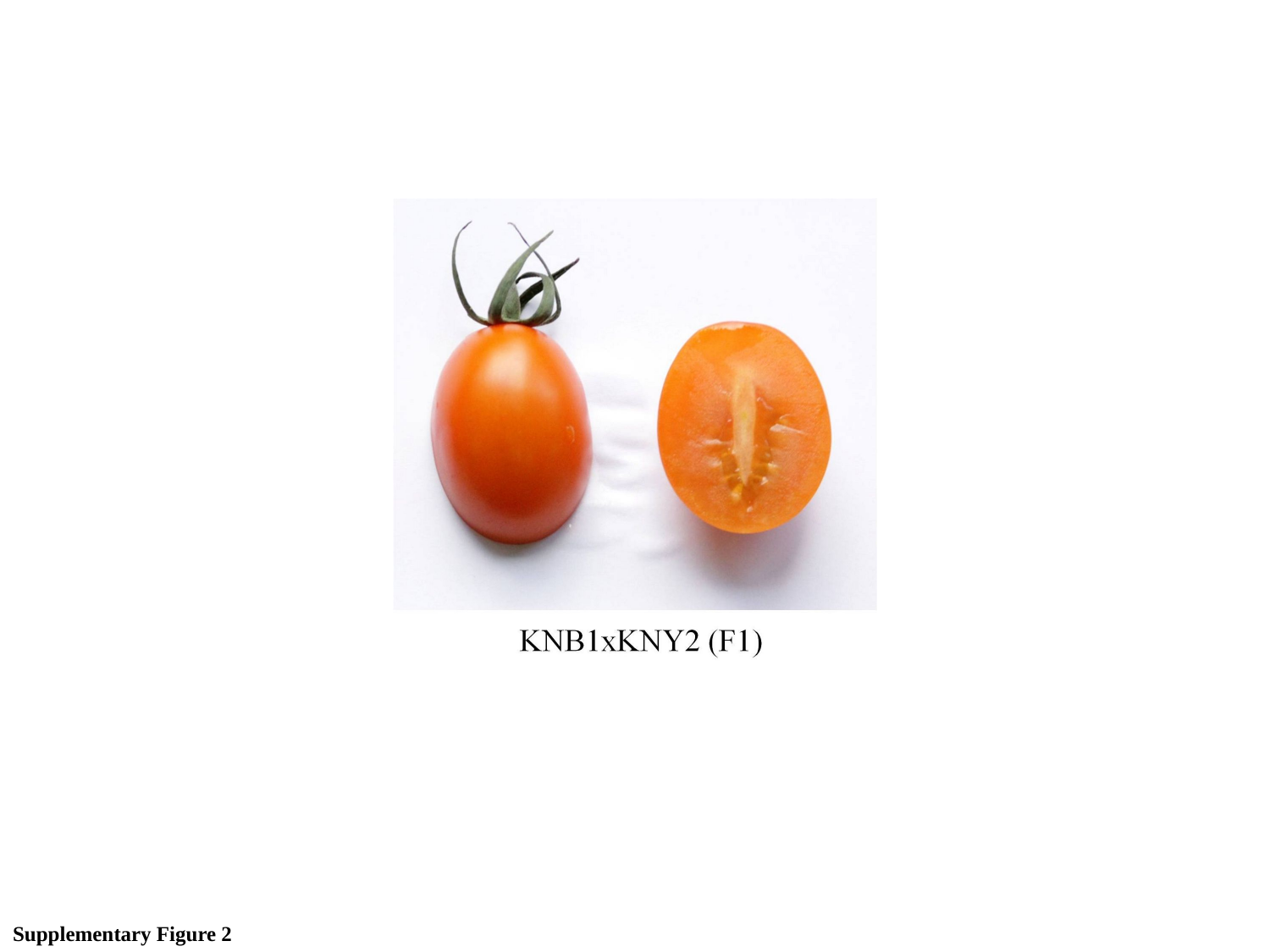

Supplementary Figure 2

## Slide 3
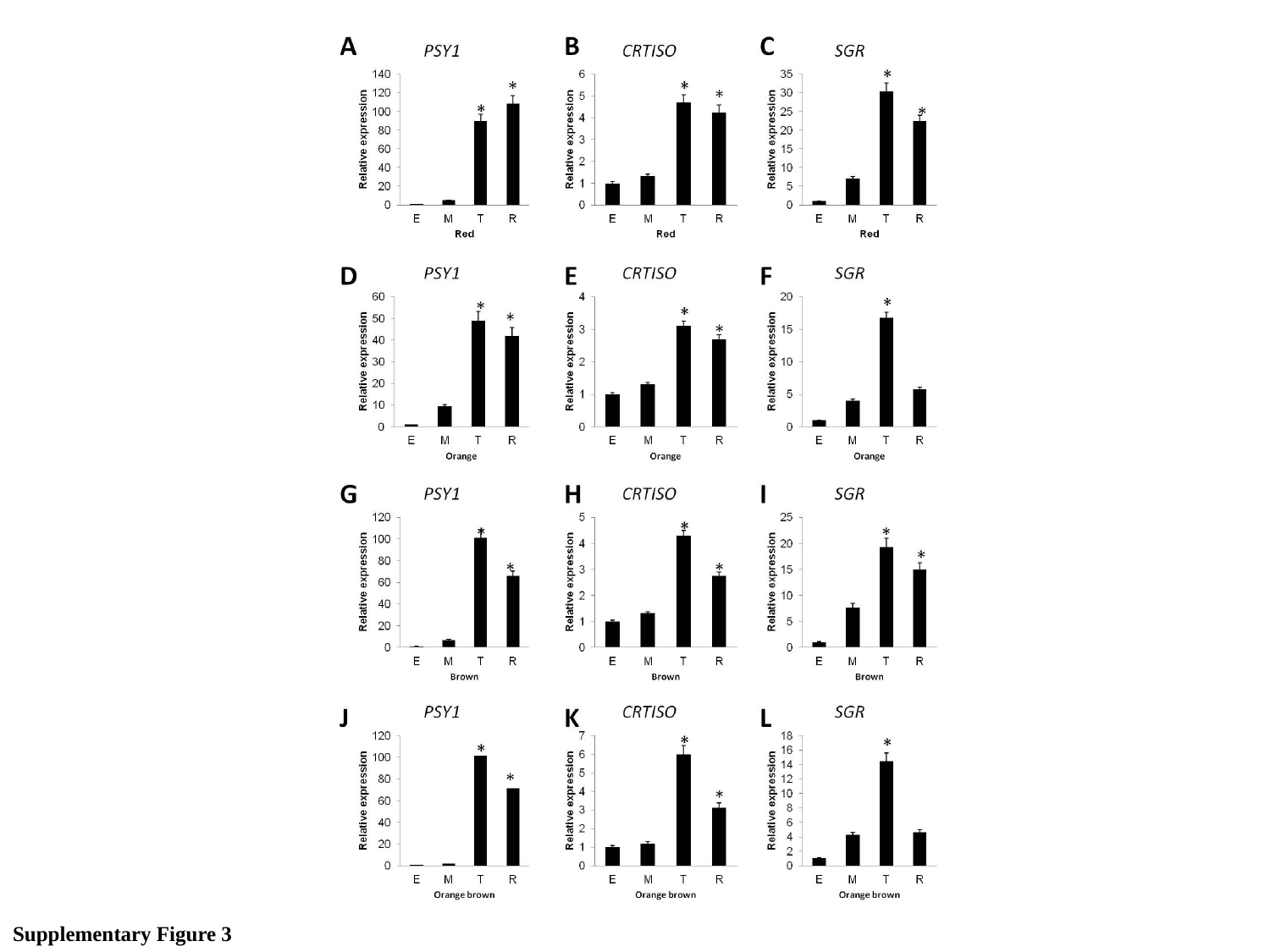

Supplementary Figure 3

Supplement: Additional file 1: Fig. S1. — Phenotype of KNB1 and KNY2 tomato fruits at various fruit ripening stages. E, early stage; M, mature stage; T, turning stage; R, ripe stage. Fig. S2. Fruit color observed in the F1 generation of a cross between KNB1 and KNY2. Fig. S3. Relative expression of PSY1 (A, D, G, J), CRTISO (B, E, H, K) and SGR (C, F, I, L) during fruit development of various inbred lines. Relative amounts of mRNA were determined by RT-qPCR after normalizing with EF1α transcript. Relative expression levels were compared to early stage (E) level (set to 1) in each F2 plant. Data represent an average ± s.e.m of three biological replicates and asterisk indicates values significantly different (p <0.05). A-C: red fruit, D-F: orange fruit, G-I: brown fruit, J-L: orange-brown fruit. E, early stage; M, mature stage; T, turning stage; R, ripe stage. (PPTX 413 kb) [file 41065_2016_23_MOESM1_ESM.pptx]
