# Supplementary material for: Molecular breeding of a novel orange-brown tomato fruit with enhanced beta-carotene and chlorophyll accumulation
Source: Hereditas. 2017 Jan 11;154:1. doi: 10.1186/s41065-016-0023-z (PMC5226094; doi:10.1186/s41065-016-0023-z)
Supplement: Additional file 2: Table S1 — Primers and probes used in this study. (DOCX 14 kb) [file 41065_2016_23_MOESM2_ESM.docx]

**Supplementary Table 1.**

| **Name** | **Sequence (5'-3')** | **Purpose** | **Reference** |
| --- | --- | --- | --- |
| **SGRF1** | AGCATCCAGGAAAGTTGCCAAGAACA | HRM primer  for SNP(C→T) | In this study |
| **SGRR1** | AGAGAGTTTCTGATAGACCTCGACTAT |  |  |
| **SGRProb4P5** | TGCAAAGAACTCCCTGTGGTAAGTTCAT |  |  |
| **CYC-BF1** | GCAAAGATTCCACTTTGTTAGCTATCTTTC | HRM primer  for SNP (G→T) | [16]  In this study |
| **CYC-BR1** | CATTATAGAGAATGTATAAGATTGATAATGG |  |  |
| **CYC-BProb1P2** | TGATTCCTCTTTAGTCCAGTA |  |  |
| **CYC-B-F** | CCATATTTAAGGGTGAATAATGCTAAA | RT-qPCR | [16] |
| **CYC-B-R** | CATTTCTCTTCCTCAATAACACTTTTC |  |  |
| **PSY1-F** | TAGATAGGTGGGAAAATAGGCTAGAA | RT-qPCR | [16] |
| **PSY1-R** | ACCGTACCAGCAACATAATAACAATA |  |  |
| **CRTISO-F** | CTGACCCAACAACTGTACATTTCCAC | RT-qPCR | [16] |
| **CRTISO-R** | TAGATGGGTTCCTCCAAAGACTTCAG |  |  |
| **SGR-F** | ATGGGAACTTTGACTACTTCTCTAG | RT-qPCR | In this study |
| **SGR-R** | ATAACCTTGCCACAGGTACTATGGA |  |  |
| **EF1α-F** | TCAGGTAAGGAACTTGAGAAGGAGCCT | RT-qPCR | [16] |
| **EF1α-R** | AGTTCACTTCCCCTTCTTCTGGGCAG |  |  |
